# PLA/PHB Blends: Biocompatibilizer Effects

**DOI:** 10.3390/polym11091416

**Published:** 2019-08-28

**Authors:** Alessandra D’Anna, Rossella Arrigo, Alberto Frache

**Affiliations:** Department of Applied Science and Technology, Viale Teresa Michel 5, 15121 Alessandria, Italy

**Keywords:** PLA, PHB, polymer blends, natural additives, HDT

## Abstract

The purpose of this work was to formulate a fully bio-based blend with superior properties, based on two immiscible polymers: polylactic acid (PLA) and poly-hydroxy butyrate (PHB). To improve the miscibility between the polymeric phases, two different kinds of compatibilizers with a different chemical structure were used, namely, an ethylene oxide/propylene oxide block copolymer in the form of flakes and a mixture of two liquid surfactants with a variable lipophilic–hydrophilic index. The morphology of the blends and their thermal, mechanical, and rheological behavior were evaluated, aiming at assessing the influence of the selected compatibilizers on the microstructure and final properties of the systems. Morphological analyses of the compatibilized blends indicated that the liquid surfactant is more effective than the solid copolymer in inducing morphology refinement, as also suggested by results coming from rheological measurements. Furthermore, thermal analyses demonstrated that the presence of both kinds of compatibilizers induced an enhancement of the crystallinity content of blends. Finally, a remarkable increase of the elastic modulus values was obtained for the compatibilized blends as compared to the pure counterparts, with a consequent significant enhancement of the HDT values.

## 1. Introduction

In the recent years, concerns related to the environmental issues have stimulated an increasing interest towards the application of biodegradable polymers derived from renewable resources as an alternative to conventional fossil fuel-based polymeric materials [1,2,3]. In this context, different biopolymers such as polylactic acid, polycaprolactone, poly (hydroxyl adipate), poly (3-hydroxybutyrate), and poly (butylene adipate-co-terephtalate) have obtained considerable attention from both academic and industrial research [4,5,6,7]. In particular, polylactic acid (PLA) is a widely studied biopolymer since, due to its intriguing characteristics, it represents a sustainable alternative to petrochemical-derived products for a large variety of applications, ranging from the industrial to biomedical field [8,9]. In fact, PLA is biodegradable and biocompatible, and its properties are very close to those of some synthetic fossil fuel-based polymers [10]. Though the values of tensile strength and elastic modulus of PLA are very similar to those of high-performance polymers [11], its brittleness and its poor barrier properties restrict its range of application [12]. To overcome the PLA drawbacks, several strategies have been proposed; one of the most successful methods is the modification of the chemical structure of the PLA chains through co-polymerization reactions of lactide with different monomers [13]. A further effective and widely practiced industrial strategy relies in the formulation of PLA-based blends through melt blending [14,15]. From an industrial point of view, blending is a much more practical and cost-effective method with respect to co-polymerization and, as a result, is the more frequently used method in the industrial sector [16,17]. In fact, following this procedure, polymeric materials with enhanced properties can be obtained, and a tuning of the mechanical and thermal properties of the blends can be achieved by adjusting the weight ratio between the selected polymers. 

Several reports are present in literature documenting the melt blending of PLA with different biodegradable or synthetic polymers, such as poly(ε-caprolactone) [18], poly(propylene) [19], poly (ethylene oxide) [20], starch [21], and poly (3-hydroxybutyrate) [22]. When the polymer selected to be blended with PLA is bio-based and/or biodegradable, a new material with low environmental impact is achieved; in this context, PLA/poly-hydroxy butyrate (PHB) blends have gained a great interest, as the combination of these two biopolymers allows obtaining new biomaterials with enhanced properties as compared to the single components, while maintaining their ecosustainability [23,24,25]. PHB is a brittle polymer, and it is fully biodegradable under both aerobic and anaerobic conditions [26]; however, its poor processability and formability are the foremost drawbacks that limit its industrial uses [27]. Zhang et al. reported the formulation of PLA/PHB blends with different weight ratios, showing that some interactions between the two polymers were established, notwithstanding their immiscibility. In particular, the blend containing 25 wt.% of PHB exhibited remarkably improved tensile properties compared with pure PLA, because of the finely dispersed PHB crystals, which acted as a filler and nucleating agent in PLA [28]. 

Recently, different approaches have been proposed to enhance the compatibility and the miscibility between PLA and PHB, including both chemical and physical methods. The first strategy refers to reactive melt-blending that can be exploited to induce some reactions between PLA and PHB chains, resulting in the in situ formation of block copolymers that act in the interfacial region between the two phases, enhancing their compatibility [29]. On the other hand, the physical methods involve the introduction of a third component able to modify the interfacial properties of the blend; more specifically, similarly to what is reported in literature for polymer-based nanocomposites [30,31], the added compatibilizers cause a reduction of the interfacial tension coefficient, with consequent formation and stabilization of a desired morphology [32]. For instance, Zembouai et al. [33] studied the effect of Sepiolite and organomodified clays on the mechanical and rheological behavior of PLA/PHB blends. Results from rheological analyses showed increased values of complex viscosity and storage modulus, attributed to the strong interactions established between the clay fillers and the polymers. 

In this work, an innovative approach involving the utilization of natural surfactants as compatibilizer for PLA/PHB blends is proposed. For this purpose, different contents of two different compatibilizing systems, either in solid or in liquid state, were melt mixed with the polymer pair and the morphology, rheological, mechanical, and thermal behaviors of the resulting systems were evaluated and compared to those of uncompatibilized blend. 

## 2. Materials and Methods

### 2.1. Materials

PLA was supplied in pellets by IngeoTM Natural Natureworks (Minnetonka, MN, USA) under the trade name PLA3251D. The polymer main properties are: density = 1.24 g/cm^3^, MFI = 33 g/10 min (210 °C, 2.16 Kg). 

PHB was manufactured by Aonilex, KANEKA Biopolymer (Osaka, Japan) under the trade name PHBX151A and commercialized in pellet form. The used PHB has the following properties: density = 1.19 g/cm^3^, MFI = 3 g/10min (165 °C, 5 Kg). 

Synperonic (Syn) is a polyalkylene oxide block copolymer in the form of flakes (at 25 °C). It is a polymeric emulsion stabilizer commercialized by CRODA under the trade name Synperonic PE_F87.

Span TM 80-LQ-(RB) is a sorbitan ester, and Tween TM 80-LQ-(CQ) is an ethoxylated sorbitan ester. Both additives are bio-based non-ionic liquid surfactants commercialized by CRODA.

Acetone from SIGMA-ALDRICH, laboratory reagent >99.5%, was used as received. 

### 2.2. Preparation of the Blends

The polymers were first dried for 5 h at 80 °C in a vacuum oven, and then the neat PLA/PHB 70/30 wt.% blend and the compatibilized systems, whose formulation is reported in Table 1, were prepared using a DSM Explore twin screw mini-extruder. The processing conditions were: T = 180 °C, screw speed = 100 RPM, time = 3 min. 

The Synperonic-containing blends were prepared using the same processing conditions, varying the additive content in a range from 0.1 to 5 wt.%. 

A further compatibilization approach was evaluated, involving the use of a liquid mixture of Span80 and Tween80. Acetone (5 mL) was used to introduce the liquid compatibilizers into the mini-extruder during the blend processing. Mixtures of Span80 and Tween80 can be prepared to achieve a requested hydrophile–lipophile balance (HLB), thus offering the possibility to adjust the polarity of the compatibilizer in order to match the polarity of the blend constituents. Knowing that HLB = 15 for Tween80 (highest hydrophobicity) and HLB = 4 for Span80 (highest hydrophilicity), it is possible to calculate the HLB index for each composition of the two emulsifiers. In this work, HLB 12 (containing 28 wt.% of Span80 and 72 wt.% of Tween80) was selected and added to the blend with a content ranging from 0.1 to 5 wt.%. HBL12-containing systems were prepared using the same processing conditions as the neat blend. 

Specimens for thermomechanical and rheological characterizations were prepared through a compression molding step at 100 bar, 190 °C for 3 min.

### 2.3. Characterizations

#### 2.3.1. Differential Scanning Calorimetry (DSC)

DSC measurements were carried out on samples of about 8 mg, placed in sealed aluminum pans, using a Q20 TA Instrument (TA Instruments Inc., New Castle, DE, USA). All the experiments were performed under dry N_2_ gas (20 mL/min). The samples were subjected to the following cycle: a heating ramp from –50 to 200 °C to erase the sample thermal history, a cooling ramp from 200 to –50 °C, and then a second heating ramp from –50 to 200 °C. All the heating/cooling ramps were performed at a scanning rate of 10 °C/min. The glass transition temperature (T_g_), crystallization temperature (T_c_), cold crystallization temperature (T_cc_), melting temperature (T_m_), and melting enthalpy (ΔH_m_) were determined from the second heating scan. The crystallinity χ of all investigated systems was evaluated as:

(1) $$\chi \;=\;\frac{\Delta \;H}{\Delta \;Hm^{0\;\;}}\cdot 100,$$

where: Δ*H* = ΔH_m_ – ΔH_cc_ (ΔH_m_ and ΔHcc being the specific melting and cold crystallization enthalpies, respectively), and Δ*Hm*^0^ is the melting enthalpy of a 100% crystalline PLA (93.0 J/g [34]).

#### 2.3.2. Thermomechanical Measurements (DMA)

DMA measurements were performed using a Q800 TA Instrument (TA Instruments Inc., New Castle, DE, USA) equipped with tension film clamps. Samples with 6 mm width × 26 mm height × 1 mm thickness were used. The temperature was varied in the range from 30 to 120 °C, applying a heating rate of 3 °C/min. The test conditions were: 1 Hz of frequency in strain-controlled mode with 15 m of amplitude, static loading of 125% of dynamic loading, and 0.01 N of preload. The heat deflection temperature (HDT) of investigated systems was calculated following the procedure exploited by Takemori [35] as the temperature at which the elastic modulus crosses the defined value of 800 MPa that correspond to an applied load of 1.82 MPa. Samples were vacuum-dried at 80 °C for 4 h before the tests. Thermomechanical tests were carried out on 3 different samples, and the error was less than 1%.

#### 2.3.3. Rheological Measurements 

Rheological measurements were performed on a rheometer ARES TA Instrument (TA Instruments Inc., New Castle, DE, USA) in parallel plate geometry, with a plate diameter of 25 mm, under nitrogen atmosphere to avoid polymer oxidative degradation. Complex viscosity and elastic and loss moduli were measured performing frequency scans from 0.1 to 100 rad/s at 190 °C. The strain was fixed at γ = 20%, which is low enough to be in the polymer linear viscoelastic regime. The typical gap between the plates imposed during the tests was 1 mm. Prior to the measurements, the samples were vacuum-dried at 80 °C for 4 h. 

#### 2.3.4. Scanning Electron Microscopy (SEM)

The surface morphology of the blends was observed using a LEO-1450VP Scanning Electron Microscope SEM (beam voltage: 20 kV). The observations were performed on the radial surfaces of the samples, obtained through fracturing in liquid nitrogen. Before the tests, the fracture surface was coated with a thin gold layer. For each micrograph, the length and diameter of the droplets of the dispersed phase were evaluated through the software Image J. This software also allows evaluating the mean, standard deviation, and minimum and maximum value of each parameter. 

## 3. Results and Discussion

### 3.1. Differential Scanning Calorimetry (DSC)

**Figure 1:**
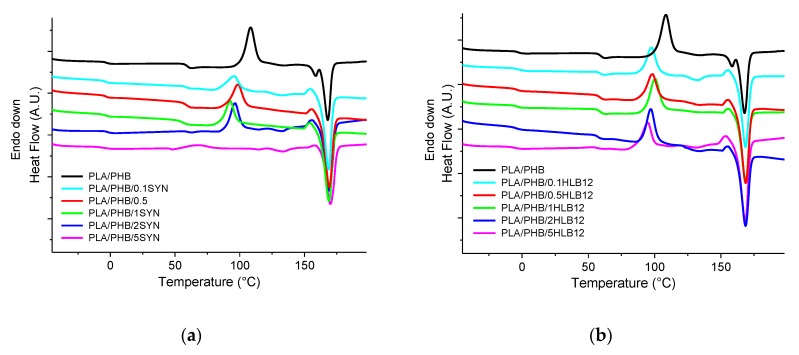
Differential scanning calorimetry (DSC) thermograms recorded during the second heating scan for (**a**) Syn- and (**b**) HBL12-containing systems, compared to that of the polylactic acid/poly-hydroxy butyrate (PLA/PHB) blend.

**Figure 2:**
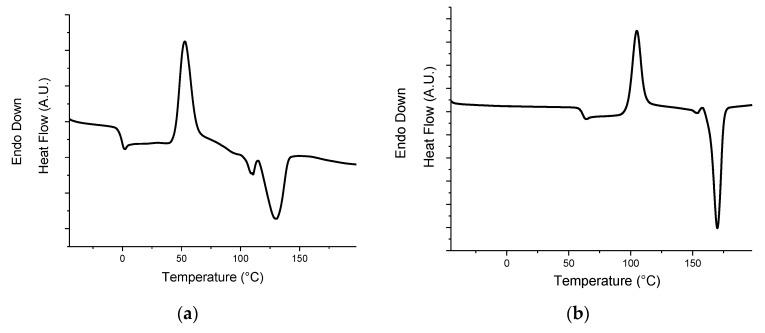
DSC thermograms recorded during the second heating scan for virgin (**a**) PHB and (**b**) PLA.

Figure 1a reports the thermograms recorded during the second heating scan of the neat blend and all the Syn-containing systems. The PLA/PHB sample shows two different T_g_ values: –4 and 59 °C, attributable to the PHB [36] and PLA [37] phases, respectively, according to the thermograms of the two virgin polymers reported in Figure 2; furthermore, an exothermic peak at 110 °C and an endothermic one at 168 °C can be observed. These peaks, in agreement with the literature [38], can be associated with the cold crystallization and the melting of the PLA phase, respectively. It is worth noting that the PHB present in the blend does not crystalize under the selected process conditions and this thermal treatment.

The presence of Syn, irrespective of its content, does not modify the T_g_ values but at the same time causes a decrease of the T_cc_ with respect to the neat blend. For the system containing 5 wt.% of Syn, the peak related to the cold crystallization is not present in the thermograms, although a crystallization peak is observed in the thermogram collected during the cooling (not reported here). Table 2 collects the main thermal properties measured during the second heating ramp. Interestingly, the presence of the Syn additive leads to a remarkable increase of the blend crystallinity degree, which passes from 0 for the neat blend to about 44% for the blend containing 5 wt.% of Syn. 

Figure 1b reports the results of the thermal characterization for the samples containing HLB12. Similarly to what was already observed for the Syn-containing blends, the T_g_ values are not affected by the presence of HLB12, whereas the T_cc_ of compatibilized samples is about 10 °C lower than that of the neat blend. Table 3 shows the ΔH_m_, ΔH_cc_, and crystallinity degree for these formulations. Once again, a significant increase of the crystallinity content can be observed as a result of the compatibilizer introduction. 

To sum up, results coming from thermal characterization indicate that the introduction of both additives causes an anticipation of the PLA cold crystallization phenomenon and a progressive increase of the crystallinity content of the blend.

### 3.2. Thermomechanical Measurements (DMA)

**Figure 3:**
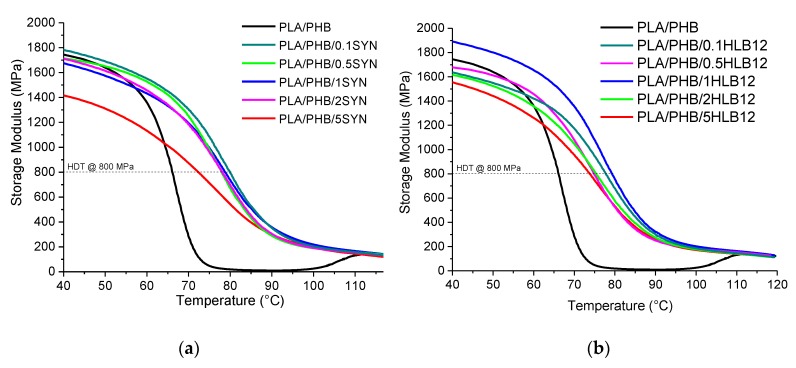
Thermomechanical measurement (DMA) traces for (**a**) Syn- and (**b**) HBL12-containing systems, compared to those of the PLA/PHB blend.

The dynamic thermomechanical analyses were used to evaluate the effect of the introduction of the selected natural compatibilizers on the mechanical properties of the PLA/PHB blends in a wide range of temperatures. In Figure 3a, the temperature dependency of the dynamic storage modulus (E’) for Syn-based blends is reported and compared to that of the neat blend. It is interesting to highlight that it is not possible to observe the glass transition of the PHB phase since the DMA tests were carried out in a room temperature. For temperatures below the glass transition of PLA, the storage modulus of the compatibilized blends is almost unchanged with respect to that of the neat blend, although a slight progressive decrease of the E’ values can be observed with increasing the additive content. This finding is more pronounced for the sample containing 5 wt.% of Syn, which exhibited a value of storage modulus significantly lower than that of PLA/PHB. At variance, the sample containing 0.1 wt.% of Syn represents an exception, since it exhibits a higher modulus than the neat blend in the described temperature range. With increasing the temperature, the elastic modulus of PLA/PHB shows a dramatic drop, due to the occurrence of the PLA glass transition; interestingly, the Syn-containing samples exhibit a less pronounced decrease of the elastic modulus, involving a consequent remarkable increase of the HDT values. This finding can be highlighted by looking at the values calculated using the procedure described in the experimental part and listed in Table 4. It is worth noting that the increase of the HDT values with respect to the neat blend is almost unaffected by the Syn amount; for this reason, the blend containing the lower amount of Syn was selected for further investigations, since this system allows obtaining superior mechanical performances, while minimizing the content of the introduced additive. In Figure 3b, the DMA curves of HLB12-based blends are reported. In the range of temperatures below the PLA glass transition temperature, the compatibilized systems show a lower modulus than the neat blend, apart from the sample containing 1.0 wt.% of HLB12, for which a higher value of E was recorded. Similarly to what was observed for the Syn-containing samples, the elastic modulus of the compatibilized blends shows a less sharp decrease as a function of temperatures, bringing about also in this case a remarkable increase of the HDT values (see values collected in Table 4). For HLB12-based systems, the values of HDT are more sensitive to the compatibilizer content with respect to the Syn-containing blends, and the maximum value of this property was reached for the sample PLA/PHB/1HLB12. As far as the HLB12-containing systems are concerned, the sample containing 1 wt.% of HLB12 was thus selected for further investigations, due to the fact that this formulation ensures improved mechanical properties, either at low or high temperatures. 

### 3.3. Rheological Measurements

**Figure 4:**
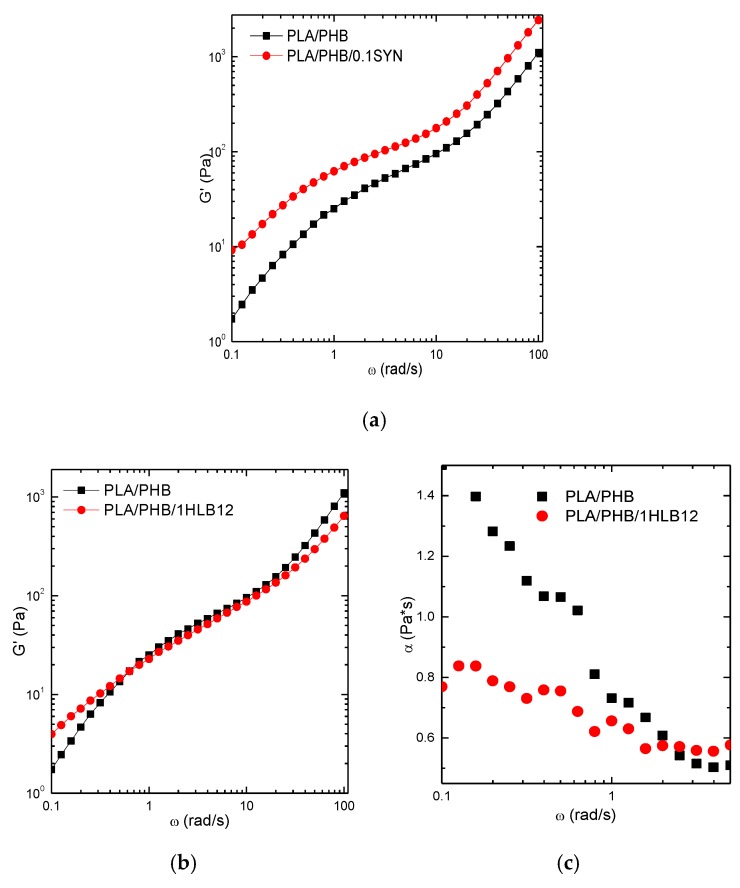
G’ curves for (**a**) PLA/PHB and PLA/PHB/0.1SYN and (**b**) for PLA/PHB and PLA/PHB/1HLB12, and (**c**) α as a function of frequency for PLA/PHB and PLA/PHB/1HLB12.

In order to study the effect of the selected natural compatibilizers on the microstructure of the blends, rheological analyses in linear dynamic shear flow were carried out. In fact, the evaluation of the rheological behavior of polymer-based blends represents an effective tool to investigate the established polymer/polymer or polymer/compatibilizer interactions, allowing to obtain important information about the morphology of the blend. Figure 4a shows the trend of the storage modulus G’ as a function of the frequency for the neat blend and the PLA/PHB/0.1SYN system. 

PLA/PHB exhibits the typical rheological response of an immiscible blend, characterized by a shoulder in the G’ curve in the low frequency region, which can be related to the relaxation of the dispersed phase that is in the form of droplets [39]. The sample containing 0.1% of Syn shows a very similar behavior in the whole investigated frequency range, but in this case, higher values of G’ are observed, as compared to the uncompatibilized blend. By contrast, significant differences emerge when comparing the rheological response of the sample containing HLB12 with that of the neat blend. In fact, as observable in Figure 4b, the presence of the liquid compatibilizer causes the disappearance of the aforementioned shoulder in the G’ trend; further, a decrease of the slope of the modulus curve in the terminal region can be observed. As widely documented in literature, this finding can be explained considering the existence of complex morphologies showing slow relaxation dynamics [40]. To further investigate this peculiar behavior, the trend of the G’ slope, for both neat and HLB12-based samples, as a function of frequency, was calculated following Equation (2):

(2) $$\alpha \;\left(\omega \right)=\frac{\alpha \log G^{\prime }}{\alpha log\omega },$$

and the obtained curves are reported in Figure 4c. 

The curve of the neat blend shows a progressive decrease of the G’ slope with increasing the frequency, reflecting the relaxation of a single dynamic specie related to the droplets constituting the dispersed phase. Differently, the curve of the compatibilized blend remains almost constant over the frequency, indicating the presence of particles of PHB of different shapes and dimensions, which are able to relax at different time scales. The structures formed by the dispersed phase, relaxing continuously over a long time, induce a continuous spectrum of relaxation times that causes the invariance of the G’ slope in the investigated frequency region. [41].

### 3.4. Scanning Electron Microscopy (SEM)

**Figure 5:**
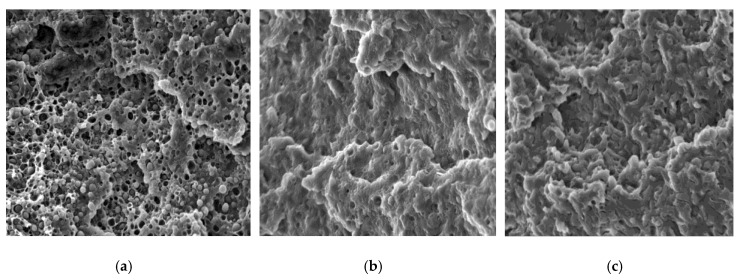
Micrographs of: (**a**) PLA/PHB, (**b**) PLA/PHB0.1SYN, and (**c**) PLA/PHB/1HLB12.

**Figure 6:**
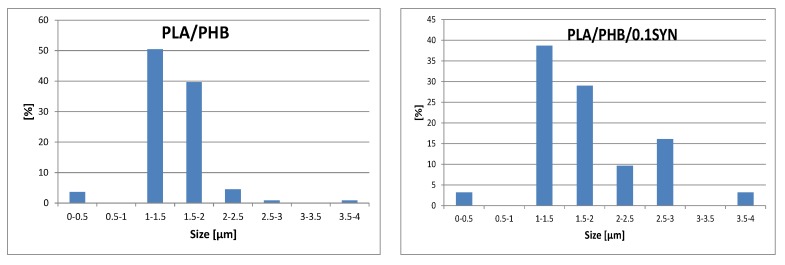
Particle size distribution of the neat blend and 0.1Syn-containing system.

In Figure 5, the typical SEM micrographs of fracture surfaces of PLA/PHB, PLA/PHB/0.1SYN, and PLA/PHB/1HLB12 systems are reported. The uncompatibilized blend shows a drop-matrix morphology, with roughly spherical PHB particles dispersed in the PLA matrix; during the fracturing, most of the PHB particles remained in the structure, while others were pulled out, leaving empty cavities on the surface. These findings, along with the observable lack of interfacial adhesion between the two phases, indicate immiscibility between PLA and PHB at the explored weight ratio. As a result of the introduction of 0.1 wt.% of Syn, different aspects can be highlighted; first, a notable reduction of the amount of pulled-out PHB particles as compared to the neat blend can be observed, as indicated by the reduced number of the empty cavities in the micrograph reported in Figure 5b. Further, a significant reduction of the average size of the dispersed particles can be noted; to quantify this finding, the mean droplet size in the neat blend and in the system containing 0.1 wt.% of Syn has been determined by the image processing software ImageJ, and the obtained results are reported in Figure 6. For the uncompatibilized blend, the main size of the PHB droplets is 1.48 μm; in the blend containing Syn, a remarkably lower number of particles can be detected.

In fact, in the PLA/PHB/0.1SYN sample, a number of particles equal to about 25% with respect of particles recognizable in the micrograph of the pure blend was calculated. This finding can be explained considering that the size of most of the PHB particles in the Syn-containing system is below the threshold of the software, at the considered magnification; furthermore, it is possible to infer that the particles of dispersed polymer are not well observable due to the more homogeneous morphology achieved upon the introduction of Syn. 

The most relevant morphological changes occurred for the sample containing HBL12; as is observable in Figure 5c, the PHB dispersed phase is hardly distinguishable from the PLA matrix and, as inferred from the rheological analyses, it appears in the form of domains with an elongated and irregular shape. 

The morphological modifications induced by the introduction of Syn and, even more, of HLB12 could be explained considering that the used natural additives were able to decrease the interfacial tension between PLA and PHB, resulting in a morphology refinement that was beneficial to improve the mechanical performances of the compatibilized blends. 

## 4. Conclusions

In this work, two different natural surfactants were exploited as compatibilizers in PLA/PHB blends. The introduction of both additives caused a significant increase of the crystallinity degree of the systems, along with a remarkable improvement of the mechanical properties, particularly at temperatures above the glass transition of the PLA major phase. Morphological observations documented a decrease of the mean particle sizes for the Syn-based blend, although the morphology of this system remains essentially drop-matrix. Otherwise, the introduction of HLB12 liquid additive induced a more evident refinement of the blend morphology, with the particles constituting the dispersed phase appearing elongated, deformed, and hardly distinguishable from the PLA matrix.

To sum up, the obtained results indicated that both selected natural surfactants are potentially suitable as compatibilizers for the PLA/HLB blends, in the framework to obtain a fully bio-based system with superior mechanical properties particularly at high temperatures, widening the typical range of application of such a kind of material, as an example, in the field of food packaging. More specifically, the introduction of HLB12 caused more remarkable morphological modifications with respect to Syn, although the drawback of using a liquid additive during the melt blending processing needs to be taken into account. By contrast, the utilization of the Syn solid surfactant presents the advantage of introducing a solid additive during the blend formulation, representing an easier and much more potentially industrially viable processing route.

## Figures and Tables

**Table 1:** Composition and code of the studied blends (all percentages are referred to as wt.%).

Composition	Code
PLA: 70% and PHB: 30%	PLA/PHB
PLA: 70%; PHB: 30% and Synperonic: 0.1%	PLA/PHB/0.1SYN
PLA: 70%; PHB: 30% and Synperonic: 0.5%	PLA/PHB/0.5SYN
PLA: 70%; PHB: 30% and Synperonic: 1%	PLA/PHB/1SYN
PLA: 70%; PHB: 30% and Synperonic: 2%	PLA/PHB/2SYN
PLA: 70%; PHB: 30% and Synperonic: 5%	PLA/PHB/5SYN
PLA: 70%; PHB: 30% and HLB12: 0.1%	PLA/PHB/0.1HLB12
PLA: 70%; PHB: 30% and HLB12: 0.5%	PLA/PHB/0.5HLB12
PLA: 70%; PHB: 30% and HLB12: 1%	PLA/PHB/1HLB12
PLA: 70%; PHB: 30% and HLB12: 2%	PLA/PHB/2HLB12
PLA: 70%; PHB: 30% and HLB12: 5%	PLA/PHB/5HLB12

**Table 2:** Thermal properties of neat blend and Syn-containing systems.

Property	PLA/PHB	0.1SYN	0.5SYN	1SYN	2SYN	5SYN
ΔH_m_ [J/g]	28	49	49	49	50	44
ΔH_cc_ [J/g]	31	15	17	23	22	0
χ [%]	0	36	34	28	30	44

**Table 3:** Thermal properties of the neat blend and HLB12-containing systems.

Property	PLA/PHB	0.1HLB12	0.5HLB12	1HLB12	2HLB12	5HLB12
ΔH_m_ [J/g]	28	48	48	46	50	56
ΔH_cc_ [J/g]	31	23	22	28	25	19
χ [%]	0	27	29	19	27	40

**Table 4:** Mechanical properties of the neat blend, Syn-, and HLB12-containing systems.

**Sample**	**PLA/PHB**	**0.1SYN**	**0.5SYN**	**1SYN**	**2SYN**	**5SYN**
Tandelta	74	86	85	85	84	84
HDT	66	80	78	79	78	72
Mod at 40 °C	1752	1800	1652	1637	1678	1375
Mod at 70 °C	266	1327	1126	1186	1215	854
Mod at 80 °C	21	806	647	745	643	545
**Sample**	**PLA/PHB**	**0.1HLB12**	**0.5HLB12**	**1HLB12**	**2HLB12**	**5HLB12**
Tandelta	74	84	82	85	83	83
HDT	66	78	75	80	75	74
Mod at 40 °C	1752	1604	1651	1871	1638	1560
Mod at 80 °C	266	1143	1083	1351	1059	967
Mod at 80 °C	21	651	534	766	586	546
